# Adiposity reduces the risk of osteoporosis in Chinese rural population: the Henan rural cohort study

**DOI:** 10.1186/s12889-020-8379-4

**Published:** 2020-03-04

**Authors:** Huiling Tian, Jun Pan, Dou Qiao, Xiaokang Dong, Ruiying Li, Yikang Wang, Runqi Tu, Tanko Abdulai, Xiaotian Liu, Jian Hou, Gongyuan Zhang, Chongjian Wang

**Affiliations:** 1 Department of Epidemiology and Biostatistics, College of Public Health, Zhengzhou University, Zhengzhou, Henan People’s Republic of China; 2 Department of Orthopaedic Surgery, The Second Affiliated Hospital and Yuying Children’s Hospital of Wenzhou Medical University, Wenzhou, Zhejiang People’s Republic of China; 3 The Second Clinical Medical school, Wenzhou Medical University, Wenzhou, Zhejiang People’s Republic of China

**Keywords:** Adiposity, Bone mineral density, Osteoporosis, Rural population

## Abstract

**Background:**

Adiposity plays a crucial role in the risk of osteoporosis. However, the impact of body fat distribution on the skeleton is contentious. The study was designed to explore the association of various adiposity indices with estimated bone mineral density (BMD) and the risk of osteoporosis based on body mass index (BMI), body fat percentage (BFP), waist circumference (WC), waist to hip ratio (WHR), waist to height ratio (WHtR), and visceral fat index (VFI).

**Methods:**

A total of 8475 subjects derived from the Henan Rural Cohort Study were analyzed. The estimated BMD of study participants were measured by calcaneal quantitative ultrasound (QUS). Linear regression and binary logistic regression were performed to estimate the association of adiposity and the outcomes.

**Results:**

The mean age of the study participants was 55.23 ± 11.09 years and 59.61% were women. The crude and age-standardized prevalence of high osteoporosis risk was 16.24 and 11.82%. Per unit increment in adiposity indices was associated with 0.005–0.021 g/cm^2^ increase in estimated BMD. The adjusted odds ratios (95% confidence interval) for high osteoporosis risk in per 1 SD increase of WC, WHR, WHtR, BMI, BFP, and VFI were 0.820 (0.748, 0.898), 0.872 (0.811, 0.938), 0.825 (0.765, 0.891), 0.798 (0.726, 0.878), 0.882 (0.800, 0.972), and 0.807 (0.732, 0.889), respectively. Stratified analyses indicated greater effects on individuals aged 55 years or older.

**Conclusions:**

The adiposity indices have an inverse association with the risk of osteoporosis among Chinese rural population, especially in the elderly.

## What is already known about this subject?

Osteoporosis leads to bone fragility, fractures with high rates of mortality, morbidity and disability. Adiposity is important in bone metabolism owing to their complicated interaction. Albeit numerous studies had explored the relationship between fat and bone, the results were conflicting. In addition, previous studies were examined their association only by a single adiposity index; it remains unclear about the overall effect of adiposity on bone health.

## What does the study add?

The results of the research indicated that increased adiposity indices were associated with higher estimated BMD and lower risk for osteoporosis in Chinese rural population aged 18–79 years, and the elderly were more likely to benefit from the protective effect adiposity indices on bone health. Furthermore, Per 1 unit increment of the adiposity indices was elevated 0.005–0.021 g/cm^2^ estimated BMD. The adjusted odds ratios for high osteoporosis risk in per 1 SD increase of WC, WHR, WHtR, BMI, BFP, and VFI were 0.820 (0.748, 0.898), 0.872 (0.811, 0.938), 0.825 (0.765, 0.891), 0.798 (0.726, 0.878), 0.882 (0.800, 0.972), and 0.807 (0.732, 0.889), respectively. These data suggest the potential role of moderate adiposity in the prevention of high osteoporosis risk.

## Mini abstract

The study was designed to explore the association of various adiposity indices on bone mineral density (BMD) and the risk of osteoporosis in China. The results showed an inverse association with high osteoporosis risk among Chinese rural population, especially in the elderly.

## Background

Osteoporosis is a disorder condition characterized by low bone mineral density (BMD) and deterioration of bone microstructure. It leads to increased bone fragility, eventually to fractures with high rates of mortality, morbidity, and disability [1]. A combined survey of the Americas and Europe reported osteoporotic fractures as the sixth burden of disability-adjusted life years (DALYs), higher than both hypertension and breast cancer [2]. Osteoporosis is becoming more common worldwide due to the aging of the global population. As the world’s most populous country, China is facing an unprecedented rate of growth in the elderly. A recent survey including 75,321 adults reported the age-standardized prevalence of osteoporosis was 6.46 and 29.13% for men and women in the elderly [3]. The number of osteoporosis fractures annually will increase to 5.99 million, and the healthcare cost is predicted to be approximately 25.43 billion dollars by 2050 [4]. Thus, it is urgent to figure out the potential mechanism of lower BMD and osteoporosis in China.

To date, very many risk factors of osteoporosis have been proposed in previous studies [5–9], including age, sex, adiposity, smoking, alcohol intake, protein and calcium intake, use of glucocorticoids, physical exercise, reproductive history and others; among which adiposity is of importance in bone metabolism because of their complicated interaction [10]. Although numerous studies have explored the relationship between fat and bone, the impact of various adiposity indices on bone mass is conflicting. The previous epidemiological studies reported that body mass index (BMI) was positively associated with BMD [11, 12], while body fat percentage (BFP) showed a negative mediator of bone mass [12]. Increased waist circumference (WC), or waist to hip ratio (WHR) and waist to height ratio (WHtR) was associated with the occurrence of low BMD or fractures [13, 14]. Besides, literature reported visceral adiposity measured by visceral fat index (VFI) was deleterious to bone microarchitecture [13, 15]. However, previous researches on the association between adiposity and bone tended to examine only by a single adiposity index in the previous studies including our research; it remains unclear that the overall effect of adiposity on bone health using different adiposity indices [11, 16, 17]. In addition, many people live in rural regions, where it is a hotspot of the incidence of osteoporosis due to poor economic status and medical resource. Therefore, the current study was designed to explore the effect of adiposity on estimated BMD and osteoporosis risk in Chinese rural population, based on WC, WHR, WHtR, BMI, BFP, and VFI.

## Methods

### Study participants

During July 2015 and September 2017, 39,259 participants aged 18–79 years old were recruited from five counties of Henan province to the Henan Rural Cohort Study and finished the baseline survey. Detailed baseline characteristics had been described in a previous study [18]. Only 8475 participants who completed the BMD measurement were included in our present study. The survey was approved by the Zhengzhou University Life Science Ethics Committee (Ethic approval code: [2015] MEC (S128)), and written informed consent was obtained from all participants.

### Data collection

Detailed information of demographic characteristics, lifestyle, the history of disease and medicine intake, and family history of disease were collected using a structured questionnaire through a face-to-face interview by the trained staff. The education level was divided into three categories: primary school or below, junior high school and high school or above. Average monthly household income was classified into “< 500 RMB”, “500–1000 RMB” and “≥1000RMB”. Marital status was grouped into living alone (unmarried/widowed/divorced) or not (married /cohabitation). Smoking status was classified into smoker (a person who smoked more than one cigarette per day in the past 6 months), ex-smoker (a person who ever smoked) and never smoker. Alcohol drinking status was categorized into drinker (a person who consumed 12 or more times in the past 1 year, whether spirits, beer, wine or other forms of alcohol beverage), ex-drinker(a person who ever drank), and never drinker. Physical activity was categorized as low, moderate and high, assessed by the International Physical Activity Questionnaire Short Form (IPAQ-SF) [19].

### Anthropometry and BMD measurements

All measurements were performed by trained investigators using calibrated equipment following the manufacturer’s instruction. Height of participants was measured twice to the nearest 0.1 cm with bare feet and in an upright posture. WC and hip circumference (HC), respectively, were measured in duplicate around the waist (halfway between the lower rib and the iliac crest) and on the widest section of the hip at a level of 0.1 cm in a horizontal plane using a standard tape measure. If two readings were more than 0.5 cm apart, a third measurement was taken, and the average of the nearest two readings was taken. WHR was calculated by the ratio of WC to HC and WHtR was WC to height. The subject was asked to stand erect on the sensing platform without shoes and holding the electrode parallel to the ground. Fat mass, BMI, BFP, and VFI were measured by the bioelectrical impendence device (V-body HBF-371; Omron, Kyoto, Japan), and its short-term in vivo coefficient of variation for the measurement of body weight, BFP, and VFI was 1%~ 3%. Fat-free mass (FFM) of the subject was body weight minus fat mass. To avoid the systematic error of excess fat mass on bone mass measured by bioelectrical impendence method, we used the corrected measurement of skeletal muscle mass in obese participants [20].

The calcaneus estimated BMD was measured using a quantitative ultrasound bone densitometer (QUS, Sahara, Hologic, USA). The bone densitometer consists of two unfocused transducers mounted coaxially on a motorized caliper. One transducer acts as a transmitter and the other as a receiver. The transducers are coupled acoustically to the heel using soft rubber pads and an oil-based coupling gel. The Sahara device measures both broadband ultrasound attenuation (BUA) and speed of sound (SOS) at a fixed region of interest in the calcaneus and the results are combined to provide an estimated BMD at heel with units of grams per square centimeter using the following equation: Estimated BMD = 0.002592*(BUA + SOS)-3.687 g/cm^2^. Subjects were asked to furl the trouser legs and sit down, the left heel was tested three times and the average reading was taken. According to the manufacturer’s data, the coefficient of variation in vivo for estimated BMD was 3%. If the subjects had a history of fractures or any bone disease in the left foot, the other heel would be measured. T-score was calculated as the difference of the subject estimated BMD and the mean BMD of healthy adults divided by the young adult standard deviation (SD). The healthy adult BMD and young adult SD were derived from a reference database of young healthy Chinese individuals provided by the manufacturer.

### Definition of obesity

According to the criteria recommended by Working Group on Obesity in China (WGOC) [21], BMI was categorized into normal (BMI < 24.0 kg/m^2^), overweight (24.0 kg/m^2^ ≤ BMI < 28.0 kg/m^2^) and obesity (BMI ≥ 28.0 kg/m^2^) for Chinese adults. In reference to the guideline of WHO [22] and International Diabetes Federation Consensus Group’s standard [23], central obesity was defined as WC equal to or greater than 90 cm for males and 80 cm for females, and further defined as WHR of over 0.90 for males and over 0.85 for females. WHtR above 0.5 was an effective indicator of health risks summarized in a review study [24]. Li L [25] proposed high-BFP with BFP ≥ 25.0% for males and BFP ≥ 35.0% for females in Chinese adults even if there was no agreement about cut-off points for the percentage of body fat that constitutes obesity in the world [26]. Owing to the inconsistent criteria in China, VFI was divided into two levels according to the cut-off value for the detection of osteoporosis (high-VFI: ≥9, low-VFI: < 9).

### Definition of osteoporosis risk

According to the given thresholds of the equipment, all individuals were classified into three general categories: low (T-score ≥ − 1), medium (− 2.5 < T-score < − 1), and high (T-score ≤ − 2.5) risk for osteoporosis. In order to define the risk for osteoporosis as a dichotomous variable, two groups were analyzed in this survey: low-medium (T-score > − 2.5) and high risk for osteoporosis (T-score ≤ − 2.5).

### Statistical analysis

Continuous variables were expressed as means ± SD and categorical variables were presented as counts with their relative frequency percentages. The comparisons of the basic characteristics between the groups were conducted using Bonferroni post hoc test in the one-way ANOVA for the continuous variables and Pearson’s chi-square test for categorical variables. The age-standardized prevalence of high risk for osteoporosis was calculated based on the sixth census data of China in 2010.

Multiple linear regression and binary logistic regression were used to evaluate the association of the six adiposity indices with estimated BMD and the risk of osteoporosis. Moderate or high correlations were found among the adiposity indices, thus only single-adiposity index models were applied (Table S1). In multivariate models, we adjusted for age, gender, FFM, education level, marital status, income level, smoking status, alcohol intake, physical activity, and dietary habits (meat and poultry intake, fresh fish, beans, vegetables and fruits).

Furthermore, we performed stratified analyses by gender (male, female), age (< 55, ≥55 years) and sensitivity analysis by excluding patients with medium osteoporosis risk. Akaike information criteria (AIC) were used to assess the relative goodness fit of the adiposity index models, where the lower the AIC, the better the fit. All statistical analyses were conducted using SAS 9.1. Statistical significance was *P* < 0.05 with 2-tailed tests.

## Results

### Characteristics of study participants

The mean age of the study participants was 55.23 ± 11.09 years and 59.61% were women. One thousand three hundred seventy-six individuals of the 8475 subjects were identified with high osteoporosis risk, and the crude and age-standardized prevalence of high osteoporosis risk was 16.24 and 11.82%. The mean estimated BMD and T-score of subjects with low or medium risk for osteoporosis were higher than individuals with high risk for osteoporosis (*P* < 0.001). Table 1 showed the general demographic and clinical characteristics of the study population. As anticipated, subjects with high osteoporosis risk were more likely to be female, older, of lower education level, living alone, more likely to smoke and drink alcohol, insufficient dietary intake of meat and poultry, fresh fish, beans, vegetables and fruits, compared to those with low or medium risk for osteoporosis (*P* < 0.05). WC, WHR, WHtR, VFI, and FFM were lower but BFP was higher in high risk osteoporosis patients.

**Table 1:** Baseline characteristics of the study population

Variables	Low risk (*n* = 3319)	Medium risk (*n* = 3780)	High risk (*n* = 1376)	*P*
Age (years), means (SD)	52.80 (11.06)^a^	55.49 (10.68)^b^	60.37 (10.43)	< 0.001
Female, n (%)	1938 (58.39)	2132 (56.40)	982 (71.37)	< 0.001
Education level, n (%)				< 0.001
Elementary school or below	1167 (35.16)	1566 (41.43)	776 (56.39)	
Junior high school	1444 (43.51)	1578 (41.75)	432 (31.40)	
High school or above	708 (21.33)	636 (16/82)	168 (12.21)	
Average monthly individual income, n (%)				< 0.001
< 500 RMB	990 (29.83)	1189 (31.46)	512 (37.21)	
500–1000 RMB	984 (29.65)	1117 (29.55)	393 (28.56)	
≥ 1000 RMB	1345 (40.52)	1474 (38.99)	471 (34.23)	
Marital status, n (%)				< 0.001
Married/cohabitating	3122 (94.06)	3448 (91.22)	1169 (84.96)	
Unmarried/divorced/widowed	197 (5.94)	332 (8.78)	207 (15.04)	
Smoker, n (%)				< 0.001
Non-smoker	2438 (73.46)	2654 (70.21)	1090 (79.21)	
Ex-smoker	263 (7.92)	353 (9.34)	103 (7.49)	
Smoker	618 (18.62)	773 (20.45)	183 (13.30)	
Drinker, n (%)				< 0.001
Non-drinker	2483 (74.84)	2775 (73.43)	1135 (82.49)	
Ex-drinker	166 (5.00)	237 (6.27)	54 (3.92)	
Drinker	669 (20.16)	767 (20.30)	187 (13.59)	
Physical activity, n (%)				< 0.001
Low	1128 (33.99)	1056 (27.94)	421 (30.60)	
Moderate	1246 (37.54)	1439 (38.07)	529 (38.44)	
High	945 (28.47)	1285 (33.99)	426 (30.96)	
Dietary habits (g/day), mean (SD)				
Meat and poultry	50.65 (84.36)^a^	47.89 (48.63)^b^	38.01 (42.94)	< 0.001
Fish	4.80 (11.71)^a^	4.94 (15.52)^b^	3.76 (8.15)	0.014
Vegetables and fruits	518.15 (275.49)^a^	517.67 (260.68)^b^	475.14 (246.01)	< 0.001
Bean	36.17 (52.19)^a^	32.95 (45.84)	30.25 (88.53)	0.003
WC (cm), mean (SD)	85.91 (10.27)^a^	84.52 (10.22)^b^	81.96 (10.46)	< 0.001
WHR, mean (SD)	0.900 (0.075)^a^	0.899 (0.082)^b^	0.886 (0.085)	< 0.001
WHtR, mean (SD)	0.535 (0.062)^a^	0.527 (0.064)^b^	0.521 (0.069)	< 0.001
BMI (kg/m^2^), mean (SD)	25.26 (3.41)^a^	24.81 (3.38)^b^	23.84 (3.47)	< 0.001
BFP, mean (SD)	29.86 (6.05)^a^	29.55 (6.30)^b^	30.98 (6.59)	< 0.001
VFI, mean (SD)	9.78 (4.59)^a^	9.48 (4.40)^b^	8.27 (4.10)	< 0.001
FFM (kg), mean (SD)	45.73 (8.17)^a^	45.03 (8.05)^b^	40.80 (7.32)	< 0.001
Estimated BMD (g/cm^2^), mean (SD)	0.55 (0.08)^a^	0.41 (0.04)^b^	0.29 (0.04)	< 0.001
T-score, mean (SD)	−0.08 (0.81)^a^	−1.76 (0.41)^b^	−2.95 (0.37)	< 0.001

^a^Low risk vs. High risk, *P* < 0.05; ^b^Medium risk vs. High risk, *P* < 0.05

Abbreviation: *RMB* Renminbi; *SD* Standard deviation, *WC* Waist circumference, *WHR* Waist to hip ratio, *WHtR* Waist to height ratio, *BMI* Body mass index, *BFP* Body fat percentage, *VFI* Visceral fat index, *FFM* Fat-free mass, *BMD* Bone mineral density

The mean and SD of estimated BMD according to the adiposity indices in different strata were presented in Table 2. People with high risk for osteoporosis had lower estimated BMD in all groups (*P* < 0.001). In the low risk for osteoporosis group, obese subjects had low estimated BMD comparing to the normal subjects in the definition of WC (*P* < 0.05). However, the distribution of estimated BMD in different strata had no significant difference in all group defined by WHR, WHtR, and VFI. Obese individuals defined by BFP had lower estimated BMD than normal subjects (*P* < 0.05) in the low-medium risk for osteoporosis groups whereas high VFI subjects had high estimated BMD than those with low VFI (*P* < 0.05) in the medium-high risk for osteoporosis groups.

**Table 2:** The distribution of estimated BMD grouped by the adiposity indices

Variables	Low risk (*n* = 3319)	Medium risk (*n* = 3780)	High risk (*n* = 1376)	*P*
WC				
Normal	0.560 ± 0.088^a^	0.408 ± 0.045	0.286 ± 0.039	< 0.001
Obesity	0.547 ± 0.079 ^a^	0.405 ± 0.043	0.286 ± 0.038	< 0.001
WHR				
Normal	0.553 ± 0.088	0.407 ± 0.044	0.286 ± 0.039	< 0.001
Obesity	0.552 ± 0.081	0.406 ± 0.043	0.286 ± 0.038	< 0.001
WHtR				
Normal	0.555 ± 0.088	0.405 ± 0.043	0.286 ± 0.037	< 0.001
Obesity	0.552 ± 0.081	0.407 ± 0.044	0.286 ± 0.039	< 0.001
BMI				
Normal/Overweight	0.553 ± 0.085	0.406 ± 0.044	0.286 ± 0.038	< 0.001
Obesity	0.548 ± 0.072	0.408 ± 0.042	0.284 ± 0.042	< 0.001
BFP				
Normal	0.555 ± 0.087 ^a^	0.405 ± 0.044 ^a^	0.288 ± 0.038	< 0.001
Obesity	0.549 ± 0.079 ^a^	0.408 ± 0.044 ^a^	0.284 ± 0.039	< 0.001
VFI				
Low	0.553 ± 0.088	0.401 ± 0.043 ^a^	0.284 ± 0.038 ^a^	< 0.001
High	0.552 ± 0.079	0.412 ± 0.044 ^a^	0.290 ± 0.038 ^a^	< 0.001

^a^The difference between the adiposity levels is significant at the 0.05 level (2-tailed)

Data are mean ± Standard Deviation. BMD was scaled to the adiposity levels (WC: < 90 cm for males and < 80 cm for females was normal; ≥90 cm for males and < 80 cm for females was obesity; WHR: < 0.9 for males and < 0.85 for females was normal; ≥0.9 for males and ≥ 0.85 for females was obesity; WHtR: < 0.5 was normal; ≥0.5 was obesity; BMI: < 28.0 kg/m^2^ was normal/overweight; ≥28.0 kg/m^2^ was obesity; BFP%: < 25% for males and < 35% for females was normal; ≥25% for males and ≥ 35% for females was obesity; VFI: < 9 was low; ≥9 was high)

Abbreviation: *WC* Waist circumference, *WHR* Waist to hip ratio, *WHtR* waist to height ratio, *BMI* Body mass index, *BFP* Body fat percentage, *VFI* Visceral fat index, *FFM* Fat-free mass, *BMD* Bone mineral density

### Associations between adiposity indices and estimated BMD

We explored the association between the six adiposity indices and estimated BMD. All six adiposity indices were positively associated with estimated BMD (*P* < 0.05). For 1 SD increment of the adiposity indices, there was 0.005–0.021 g/cm^2^ estimated BMD increase (Table 3). The associations were likely to be stronger for VFI and BMI. More robust associations were found in the sensitivity analysis after excluded the subjects with medium risk for osteoporosis (Table S3). When stratified by age and gender, the relationship between the adiposity indices and estimated BMD remained (Table 3). Stratified analyses showed that the effect estimates were greater in the elderly subjects, however, there was no statistical significance grouped by gender.

**Table 3:** Adjusted estimates for BMD measurement per SD increase of the adiposity indices^a^

	Total	Male	Female	< 55 years old	≥55 years old
WC	0.009 (0.006, 0.013)	0.012 (0.006, 0.018)	0.011 (0.006, 0.016)	0.005 (−0.001, 0.010)	0.008 (0.003, 0.013)
WHR	0.005 (0.002, 0.008)	0.006 (0.001, 0.011)	0.008 (0.004, 0.012)	0.001 (−0.004, 0.005)	0.006 (0.002, 0.010)
WHtR	0.011 (0.008, 0.014)	0.015 (0.010, 0.020)	0.011 (0.008, 0.016)	0.009 (0.004, 0.014)	0.009 (0.005, 0.013)
BMI	0.020 (0.017, 0.024)	0.025 (0.018, 0.031)	0.019 (0.014, 0.024)	0.018 (0.012, 0.023)	0.017 (0.012, 0.022)
BFP	0.011 (0.007, 0.015)	0.012 (0.007, 0.018)	0.013 (0.008, 0.019)	0.008 (0.002, 0.013)	0.009 (0.004, 0.015)
VFI	0.021 (0.017, 0.025)	0.022 (0.017, 0.027)	0.019 (0.014, 0.025)	0.017 (0.011, 0.022)	0.013 (0.008, 0.018)

^a^Data are β (95% Confidence Interval). β indicates partial regression coefficient. Estimates were scaled to per SD for the adiposity indices (10.37 cm for WC, 0.080 for WHR, 0.064 for WHtR, 3.44 kg/m^2^ for BMI, 6.27% for BFP, and 4.46 for VFI)

Data are adjusted for age, gender, FFM, education level, marital status, income level, smoking, alcohol intake, physical activity and dietary (meat and poultry, fresh fish, beans, vegetables and fruits)

Abbreviation: *WC* Waist circumference, *WHR* Waist to hip ratio, *WHtR* Waist to height ratio, *BMI* Body mass index, *BFP* Body fat percentage, *VFI* Visceral fat index, *FFM* Fat-free mass, *BMD* Bone mineral density

### Associations between adiposity indices and osteoporosis risk

For the high risk for osteoporosis patients per 1 SD increase in the adiposity indexes, we observed inverse association with all the six adiposity indexes (Table 4). Per unit increment of WC, WHR, WHtR, BMI, BFP, and VFI were associated with a 18.0, 12.8, 17.5, 20.2,11.8, and 19.3% decreased high risk to low-medium risk for osteoporosis, respectively. That was to say, adiposity might protect against high risk for osteoporosis. Moreover, the model including WHtR had a good fit (AIC = 6751.1), after adjusting for all potential confounders (Table S3). To the robustness, we also found people who were obese in the different definition of the adiposity indices, had a lower risk for osteoporosis, comparing to those with normal adiposity indices (Table 5). After we removed the medium risk for osteoporosis subjects, the inverse association did not change significantly for the adiposity indices (Table S4). Stratified analyses by sex and age, presented the decrease in osteoporosis risk seemed to be greater for individuals who were elder (aged ≥55 years) (Table 4, Table 5).

**Table 4:** Adjusted OR for high osteoporosis risk associated with a SD increase of the adiposity indices

	Total	Male	Female	< 55 years old	≥55 years old
WC	0.820 (0.748, 0.898)	0.820 (0.695, 0.968)	0.768 (0.686, 0.860)	0.849 (0.723, 0.996)	0.870 (0.781, 0.970)
WHR	0.872 (0.811, 0.938)	0.912 (0.795, 1.046)	0.817 (0.748, 0.892)	0.952(0.842, 1.076)	0.861 (0.789, 0.939)
WHtR	0.825 (0.765, 0.891)	0.793 (0.686, 0.917)	0.796 (0.726, 0.872)	0.821 (0.716, 0.942)	0.868 (0.794, 0.948)
BMI	0.798 (0.726, 0.878)	0.675 (0.563, 0.810)	0.820 (0.731, 0.919)	0.800 (0.678, 0.943)	0.854 (0.762, 0.956)
BFP	0.882 (0.800, 0.972)	0.808 (0.696, 0.938)	0.889 (0.780, 1.014)	0.926 (0.789, 1.088)	0.938 (0.835, 1.053)
VFI	0.807 (0.732, 0.889)	0.739 (0.632, 0.864)	0.829 (0.731, 0.940)	0.825 (0.692, 0.983)	0.898 (0.804, 1.004)

Data are OR (95% Confidence Interval) for developing high osteoporosis risk in per SD increment of the six adiposity indices. OR was scaled to the SD for each adiposity index (10.37 cm for WC, 0.080 for WHR, 0.064 for WHtR, 3.44 kg/m^2^ for BMI, 6.27% for BFP, and 4.46 for VFI)

Data are adjusted for age, gender, FFM, education level, marital status, income level, smoking, alcohol intake, physical activity and dietary (meat and poultry, fresh fish, beans, vegetables and fruits)

Abbreviation: *OR* Odds ratio, *WC* Waist circumference, *WHR* Waist to hip ratio, *WHtR* Waist to height ratio, *BMI* Body mass index, *BFP* Body fat percentage, *VFI* Visceral fat index, *FFM* Fat-free mass

**Table 5:** Associations of obesity in different definitions with high osteoporosis risk

	Total	Male	Female	< 55 years old	≥55 years old
WC	0.744 (0.636, 0.871)	0.926 (0.684, 1.255)	0.644 (0.534, 0.777)	0.843 (0.649, 1.096)	0.750 (0.617, 0.912)
WHR	0.716 (0.622, 0.825)	0.842 (0.659, 1.075)	0.617 (0.517, 0.735)	0.772 (0.615, 0.968)	0.715 (0.597, 0.856)
WHtR	0.672 (0.580, 0.779)	0.716 (0.557, 0.918)	0.609 (0.506, 0.733)	0.742 (0.584, 0.942)	0.684 (0.568, 0.823)
BMI	0.934 (0.756, 1.149)	1.234 (0.828, 1.839)	0.816 (0.638, 1.042)	0.662 (0.452, 0.971)	1.133 (0.883, 1.455)
BFP	0.973 (0.852, 1.112)	0.821 (0.652, 1.034)	0.999 (0.847, 1.179)	1.013 (0.792, 1.295)	1.021 (0.874, 1.193)
VFI	0.775 (0.666, 0.901)	0.596 (0.455, 0.781)	0.827 (0.688, 0.993)	0.745 (0.565, 0.982)	0.870 (0.729, 1.040)

Data are OR (95% Confidence Interval) for developing high osteoporosis risk of obesity/high vs. normal/normal and overweight/low in different obesity definitions. (WC: < 90 cm for males and < 80 cm for females was normal; ≥90 cm for males and < 80 cm for females was obesity; WHR: < 0.9 for males and < 0.85 for females was normal; ≥0.9 for males and ≥ 0.85 for females was obesity; WHtR: < 0.5 was normal; ≥0.5 was obesity; BMI: < 28.0 kg/m^2^ was normal/overweight; ≥28.0 kg/m^2^ was obesity; BFP%: < 25% for males and < 35% for females was normal; ≥25% for males and ≥ 35% for females was obesity; VFI: < 9 was low; ≥9 was high)

Data are adjusted for age, gender, FFM, education level, marital status, income level, smoking, alcohol intake, physical activity and dietary (meat and poultry, fresh fish, beans, vegetables and fruits)

Abbreviation: *OR* Odds ratio, *WC* Waist circumference, *WHR* Waist to hip ratio, *WHtR* Waist to height ratio, *BMI* Body mass index, *BFP* Body fat percentage, *VFI* Visceral fat index, *FFM* Fat-free mass

## Discussion

To our knowledge, this is the first study to explore the association of the six anthropometric adiposity indices with estimated BMD and the risk of osteoporosis in a large epidemiological study. We found that increment in all adiposity indices was significantly associated with higher estimated BMD and lower risk for osteoporosis. The association was independent of established risk factors, including age, gender, FFM, education level, marital status, income level, smoking, alcohol intake, physical activity, and dietary factors. In addition, we observed that the elderly subjects appeared to be more predisposed to the fat-protect-bone effects.

### Comparison with other studies

Several previous studies investigated the associations between adiposity indices and BMD or osteoporosis, but the findings were inconsistent [11–13, 18]. In agreement with our findings, a cohort of 16,500 women aged 50 years and older found high BMD in the highest BMI values (≥30 kg/m^2^) [11]. Meanwhile, a meta-analysis included 60,000 subjects with over 250,000 person-years reported that the risk of hip fractures with a BMI of 20 kg/m^2^ was increased two-fold comparing with a BMI of 25 kg/m^2^ [16]. Although it was widely accepted obesity was protective against the risk of osteoporosis, newer studies suggested increasing adiposity might be harmful to bone microarchitecture [13, 15, 27, 28]. A study conducted by Chin et al. discovered BFP was inversely associated with bone health although BMI was positively associated with bone status [27]. The reason for the contradictory results with our research might be the different inclusion criteria of covariates in the models. Furthermore, the adverse association of visceral fat with bone structure and strength was found where computed tomography (CT) was used to measure fat mass and bone health [15]. However, visceral fat had a positive association with estimated BMD in our study. This could be attributed to the nature of CT which could reflect not only bone microstructure but bone density.

### Biological mechanism of obesity and osteoporosis

Although the underlying biological mechanisms whereby obesity may protect against osteoporosis are not entirely clear, several potential biological pathways have been proposed. One straightforward explanation is that obese individuals have higher BMD in order to withstand the greater force of higher weight (i.e. lean mass and fat mass) mechanical loading on the bones than their normal-weight counterparts [29, 30]. It was reported lean mass exerts a greater protective effect on bone mass than fat mass [30]. The potential mechanism of muscle-protected bone is that bone strength is enhanced and mechanoreceptors on bone cells can promote the remodeling of cortical bone, thereby increasing bone mass and strength during muscle contraction [31]. Besides, the protective effect has been found even at non-weight-bearing bone sites [32]. Thus, another explanation has been proposed that some adipokines such as adiponectin [33], resistin [34], secreted by adipose tissue stimulate bone formation by inducing the differentiation of mesenchymal stem cells (MSCs) to osteoblast and increasing osteoblast proliferation. The positive associations of obesity with BMD in our study support these mechanisms and provide support for the obesity effects on osteoporosis.

### Age-stratification descriptive and potential biological mechanism

In an age-stratified analysis, we observed that the association of obesity and estimated BMD was stronger among the elder subgroup (≥55 years). These findings were partially consistent with the previous epidemiological studies which explore the effects of obesity on BMD [27, 35–37]. For example, a large cohort study [35] reported that BMD at the lumbar spine among premenopausal women was 1.5-fold higher than the postmenopausal women. Another large study [37] reported that in the older group lower BMI levels were associated with decreased BMD levels at the femoral site, and the effect of BMI on BMD was significantly enhanced with aging at the hip. A recent study [27] included younger and older subjects simultaneously, showed that the older individuals had stronger effects of adiposity on BMD than the younger individuals, without any gender interaction. Although the potential biological mechanism that obesity may protect age-related bone loss is not fully understood, a plausible explanation for our results is that obese subjects had to withstand stronger mechanical loading to the bone with aging, and the effect surpasses the accelerated bone loss compared to the young subjects.

### Strengths and limitations

One noticed strength of our research is that it can compare the magnitude of the impact of those adiposity indices on estimated BMD and the risk of osteoporosis. The partial regression coefficients of the six adiposity indices, in their respective SD, provide a reliable comparison for the comparable increments [38]. Although this was a large cross-sectional study to assess the relationship between obesity and the risk of osteoporosis, some limitations should be considered as well. First, dual-energy X-ray (DXA), the gold standard method for measuring BMD was not used in the study, while QUS used in the research might reduce the accuracy of BMD measurement. However, QUS for prediction of BMD reported good specificity for the indication for osteoporosis by DXA [39]. Besides, it is convenient, suitable, and available for us to measure BMD than DXA in the large-scale study. Second, some adiposity indices were measured by bioelectrical impendence methods in our research, which was less accurate, comparing to the gold standard, but this method was widely used in previous studies [40, 41]. Third, this was an observational study. As same to all observational studies, some unmeasured confounding possibly existed. However, our results were robust as if we adjusted for numerous potential confounding including lifestyle factors, exercise, and dietary factors. Moreover, the unraveling reverse causality couldn’t be ruled out because of the survey based on a cross-sectional study. But lots of prospective studies and meta-analysis were conducted prior to this study, which were parallel to our findings.

## Conclusions

In conclusion, the study indicated that increased adiposity indices were associated with higher estimated BMD and lower risk of osteoporosis in Chinese rural adults. In addition, the elderly were more vulnerable to be protective for poor bone health. These data suggest a potential role of moderate adiposity in the prevention of high osteoporosis risks. Considering the limitations of our study, more longitudinal researches are needed to evaluate the effects of adiposity on bone health.

## Supplementary information

**Additional file 1: Table S1.** Pairwise correlations between the adiposity indices. **Table S2.** Adjusted estimates for BMD per SD increase of the adiposity indices after removed. **Table S3.** Adjusted OR high osteoporosis risk associated with a SD increase of the adiposity indices after excluding medium osteoporosis risk patients. **Table S4.** Odd ratios for osteoporosis with a SD increase of the adiposity indices.

## Data Availability

The data used in this study are available and will be provided by the corresponding author on a reasonable request.

## Abbreviations

AIC: Akaike information criteria
BFP: Body fat percentage
BMD: Bone mineral density
BMI: Body mass index
BUA: Broadband ultrasound attenuation
DXA: Dual-energy X-ray
FFM: Fat-free mass
HC: Hip circumference
MSCs: Mesenchymal stem cells
SD: Standard deviation
SOS: Speed of sound
VFI: Visceral fat index
WC: Waist circumference
WHR: Waist to hip ratio
WHtR: Waist to height ratio
