# Supplementary material for: Adiposity reduces the risk of osteoporosis in Chinese rural population: the Henan rural cohort study
Source: BMC Public Health. 2020 Mar 4;20:285. doi: 10.1186/s12889-020-8379-4 (PMC7057635; doi:10.1186/s12889-020-8379-4)
Supplement: Supplementary file 1 — Additional file 1: Table S1. Pairwise correlations between the adiposity indices. Table S2. Adjusted estimates for BMD per SD increase of the adiposity indices after removed. Table S3. Adjusted OR high osteoporosis risk associated with a SD increase of the adiposity indices after excluding medium osteoporosis risk patients. Table S4. Odd ratios for osteoporosis with a SD increase of the adiposity indices. [file 12889_2020_8379_MOESM1_ESM.docx]

**Table S1. Pairwise correlations between the adiposity indices**

| Adiposity index | Pearson correlation coefficients | | | | | | |
| --- | --- | --- | --- | --- | --- | --- | --- |
|  | WC | WHR | WHtR | BMI | BFP | VFI | FFM |
| WC | 1 |  |  |  |  |  |  |
| WHR | 0.798^*^ | 1 |  |  |  |  |  |
| WHtR | 0.913^*^ | 0.770^*^ | 1 |  |  |  |  |
| BMI | 0.838^*^ | 0.551^*^ | 0.840^*^ | 1 |  |  |  |
| BFP | 0.417^*^ | 0.291^*^ | 0.635^*^ | 0.545^*^ | 1 |  |  |
| VFI | 0.806^*^ | 0.584^*^ | 0.715^*^ | 0.850^*^ | 0.234^*^ | 1 |  |
| FFM | 0.556^*^ | 0.329^*^ | 0.218^*^ | 0.478^*^ | -0.365^*^ | 0.651^*^ | 1 |

*Correlation is significant at the 0.01 level (2-tailed).

Abbreviation: WC, waist circumference; WHR, waist to hip ratio; WHtR, waist to height ratio; BMI, body mass index; BFP, body fat percentage; VFI, visceral fat index; FFM, fat-free mass.

**Table S2. Adjusted estimates for BMD per SD increase of the adiposity indices after removed medium osteoporosis risk patients.**

|  | β (95%CI) (After excluding) | β (95%CI) (Before excluding) |
| --- | --- | --- |
| WC | 0.010 (0.004, 0.016) | 0.009 (0.006, 0.013) |
| WHR | 0.007 (0.002, 0.011) | 0.005 (0.002, 0.008) |
| WHtR | 0.013 (0.008, 0.018) | 0.011 (0.008, 0.014) |
| BMI | 0.026 (0.020, 0.032) | 0.020 (0.017, 0.024) |
| BFP | 0.011 (0.005, 0.017) | 0.011 (0.007, 0.015) |
| VFI | 0.026 (0.021, 0.032) | 0.021 (0.017, 0.025) |

Data are adjusted for age, gender, FFM, education level, marital status, income level, smoking, alcohol intake, physical activity and dietary (meat and poultry, fresh fish, beans, vegetables and fruits).

Abbreviation: CI: confidence interval; WC, waist circumference; WHR, waist to hip ratio; WHtR, waist to height ratio; BMI, body mass index; BFP, body fat percentage; VFI, visceral fat index; FFM, fat-free mass.

**Table S3. Adjusted OR high osteoporosis risk associated with a SD increase of the adiposity indices after excluding medium osteoporosis risk patients.**

|  | OR (95%CI) (After excluding) | OR (95%CI) (Before excluding) |
| --- | --- | --- |
| WC | 0.704 (0.632, 0.784) | 0.820 (0.748, 0.898) |
| WHR | 0.834 (0.768, 0.906) | 0.872 (0.811, 0.938) |
| WHtR | 0.730 (0.668, 0.798) | 0.825 (0.765, 0.891) |
| BMI | 0.723 (0.649, 0.806) | 0.798 (0.726, 0.878) |
| BFP | 0.817 (0.732, 0.912) | 0.882 (0.800, 0.972) |
| VFI | 0.726 (0.650, 0.810) | 0.807 (0.732, 0.889) |

Data are OR (95% Confidence Interval) for developing high osteoporosis risk in per SD increment of the six adiposity indices. OR was scaled to the SD for each adiposity index (10.37cm for WC, 0.080 for WHR, 0.064 for WHtR, 3.44 kg/m^2^ for BMI, 6.27% for BFP, and 4.46 for VFI)

Data are adjusted for age, gender, FFM, education level, marital status, income level, smoking, alcohol intake, physical activity and dietary (meat and poultry, fresh fish, beans, vegetables and fruits).

Abbreviation: OR, odd ratio; CI: confidence interval; WC, waist circumference; WHR, waist to hip ratio; WHtR, waist to height ratio; BMI, body mass index; BFP, body fat percentage; VFI, visceral fat index; FFM, fat-free mass.

**Table S4. Odd ratios for osteoporosis with a SD increase of the adiposity indices.**

|  | Model 1 | |  | Model 2 | |
| --- | --- | --- | --- | --- | --- |
|  | OR (95%CI) | AIC |  | OR (95%CI) | AIC |
| WC | 0.635 (0.591, 0.683) | 6877.0 |  | 0.820 (0.748, 0.898) | 6757.5 |
| WHR | 0.718 (0.667, 0.772) | 6756.6 |  | 0.872 (0.811, 0.938) | 6756.6 |
| WHtR | 0.650 (0.605, 0.698) | 6890.2 |  | 0.825 (0.765, 0.891) | 6751.1 |
| BMI | 0.645 (0.600, 0.695) | 6873.3 |  | 0.798 (0.726, 0.878) | 6759.2 |
| BFP | 0.679 (0.614, 0.750) | 6886.2 |  | 0.882 (0.800, 0.972) | 6774.5 |
| VFI | 0.629 (0.580, 0.683) | 6820.0 |  | 0.807 (0.732, 0.889) | 6761.8 |

Data are OR (95% Confidence Interval) for developing high osteoporosis risk in per SD increment of the six adiposity indices. OR was scaled to the SD for each adiposity index (10.37cm for WC, 0.080 for WHR, 0.064 for WHtR, 3.44 kg/m^2^ for BMI, 6.27% for BFP, and 4.46 for VFI)

Model 1: adjusted for age, gender.

Model 2: adjusted for age, gender, FFM, education level, marital status, income level, smoking, alcohol intake, physical activity and dietary (meat and poultry, fresh fish, beans, vegetables and fruits).

Abbreviation: OR, odd ratio; WC, waist circumference; WHR, waist to hip ratio; WHtR, waist to height ratio; BMI, body mass index; BFP, body fat percentage; VFI, visceral fat index; FFM, fat-free mass.
